# A Qualitative Evaluation of COVID-19 Preventative Response Activities in South Kivu, Democratic Republic of the Congo

**DOI:** 10.3390/ijerph192013424

**Published:** 2022-10-17

**Authors:** Matthew A. Aubourg, Lucien Bisimwa, Jean Claude Bisimwa, Presence Sanvura, Camille Williams, Raissa Boroto, Claude Lunyelunye, Jessy Timsifu, Brigitte Munyerenkana, Kelly Endres, Peter J. Winch, Justin Bengehya, Ghislain Maheshe, Cirhuza Cikomola, Alain Mwishingo, Christine Marie George

**Affiliations:** 1 Department of International Health, Johns Hopkins Bloomberg School of Public Health, Baltimore, MD 21205, USA; 2 Center for Tropical Diseases & Global Health, Université Catholique de Bukavu, Bukavu B.P 265, Democratic Republic of the Congo; 3 Bureau de l’Information Sanitaire, Surveillance Epidémiologique et Recherche Scientifique, Division Provinciale de la Santé Sud Kivu, Ministère de la Santé, Bukavu B.P 265, Democratic Republic of the Congo; 4 Faculty of Medicine, Université Catholique de Bukavu, Bukavu B.P 265, Democratic Republic of the Congo

**Keywords:** Democratic Republic of the Congo, South Kivu, COVID-19, semi-structured interviews, qualitative research, program evaluation, key informants, community members

## Abstract

Objective: In this evaluation of COVID-19 preventative response programs in South Kivu, Democratic Republic of the Congo (DRC), we aimed to explore community understandings of COVID-19, assess operational successes and challenges of COVID response activities, and identify barriers to practicing COVID-19 preventative behaviors. Methods: Thirty-one semi-structured interviews were conducted from April to September 2021 in South Kivu, DRC, with community members (*n* = 16) and programmatic stakeholders (*n* = 15) (healthcare providers, government officials, and developmental and NGO staff engaged in COVID-19 response). Findings: Most community members were aware of COVID-19 and its global burden, but few were aware of local transmission in their area. Some community members attributed COVID-19 to actions of malevolent neighbors, miasma (“bad air”), or spirits. Awareness of COVID-19 preventative measures was widespread, largely because of radio and TV health promotion programs. Community members and programmatic stakeholders both said community-level non-compliance to COVID-19 preventative measures was high despite high awareness of preventative methods. Community members expressed concern that face masks distributed as part of preventative programs contained the COVID-19 virus. Programmatic stakeholders emphasized the need for broader health system strengthening with improved coordination, provision of resources to health facilities at the provincial level, and prioritization of research. Lessons learned from addressing Ebola were leveraged for COVID-19 health promotion, rapid training of healthcare personnel, and surveillance. Conclusions: Community-informed approaches are needed for effective COVID-19 preventative response programs in South Kivu, DRC. Our study identified successes and challenges in COVID-19 response activities. Future research should assess the effectiveness of integrating preventive programs with COVID-19 vaccination efforts.

## 1. Introduction

The first confirmed COVID-19 case was reported to have reached sub-Saharan Africa in February 2020 [1]. High levels of poverty, overburdened healthcare systems and facilities, and limited personal protective equipment (PPE), laboratory, and surveillance resources were anticipated to exacerbate the COVID-19 disease burden in this region [2,3]. However, despite initial projections, the relative impact of the pandemic in case numbers and deaths was low in relation to other areas of the world such as in Europe and the United States [4,5]. Reports suggest that underreporting due to a lack of diagnostic testing may be responsible for lower recorded case numbers [2,6]. Other reports suggest relatively low population density, a younger population demographic profile than other regions, and a low urbanization rate as factors contributing to lower case numbers [4].

Previous experiences responding to infectious diseases such as Ebola virus disease (EVD) have also equipped sub-Saharan Africa health systems with lessons to facilitate their COVID-19 response efforts [7,8,9,10,11]. Studies in South Africa have shown high knowledge of COVID-19 and preventative measures (i.e., masking, handwashing, physical distancing) [12]. However, higher knowledge was not always associated with greater compliance with protective behaviors [13,14]. In Zimbabwe and the Democratic Republic of the Congo (DRC), common barriers to compliance with preventative measures included living and working in crowded areas, local understandings of COVID-19, and misinformation through social media [15,16]. Previous studies in the sub-Saharan Africa region have recommended tailoring health communication to the local context [17,18,19], prioritizing trust-building between communities and the Ministries of Health (MoH) [20,21], and ensuring adequate provision of hygiene and PPE materials for effective COVID-19 response programs [15,22]. 

In the DRC, 92,934 confirmed COVID-19 cases and 1443 related deaths have been reported to the World Health Organization as of October 2022 [23]. Countrywide vaccination is currently low at 4.18 total doses administered per 100 individuals [23]. Continued widespread civil and political unrest also serves as a challenge to a coordinated COVID-19 response in the DRC, particularly in North and South Kivu in the east [24,25]. South Kivu has an estimated population of approximately 6 million and is largely rural outside of major urban city centers such as Bukavu. The South Kivu has the highest income inequality of the DRC provinces with a Gini coefficient of 0.42 [26]. Levels of fertility (7.7%) and female employment (77%) exceed that of other provinces, while female and male secondary education completion fall behind (3.1% and 8.5%, respectively). DRC MoH in South Kivu partnered with external developmental and non-governmental organizations to coordinate and implement COVID-19 response activities. COVID-19 health promotion has been communicated through radio and television (TV) advertisements, and community megaphone announcements throughout South Kivu (personal communication, Dr. Lucien Bisimwa, 21 June 2022). Some phone companies (e.g., Orange and Airtel) have included messages about hygiene practices, physical distancing, and mask wearing in their ringtones. In the city of Bukavu, communal handwashing stations—alongside posters instructing about when and how to wash hands with soap or to use chlorinated water—have been placed in areas of high foot traffic such as markets, health facilities, and schools (George et al., 2022, In press) [27]. The government implemented a countrywide lockdown of schools and some workplaces, restricted movements between provinces, and closed international borders from April 2020 to September 2020. After the second wave of COVID-19 in the beginning of 2021, public health authorities also issued a second police-enforced masking mandate and curfew [28,29].

To date, limited work has been conducted to evaluate the effectiveness of COVID-19 response and control programs in the DRC. In our qualitative evaluation of COVID-19 preventative response programs in South Kivu, we investigated the perspectives and experiences of community members and programmatic stakeholders (i.e., healthcare providers, government officials, and developmental and NGO staff engaged in the COVID-19 response) through semi-structured interviews. 

## 2. Materials and Methods

### 2.1. Site Description

**Figure 1:**
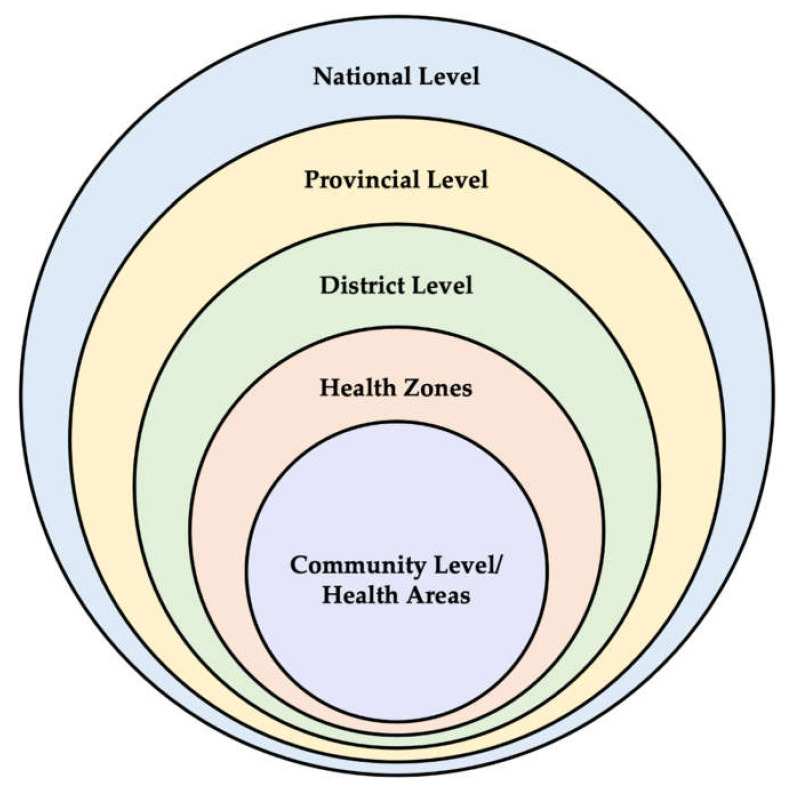
Schematic of health system levels in South Kivu.

This qualitative evaluation of COVID-19 preventative response programs was conducted in Bukavu, DRC. Bukavu is the capital city of the South Kivu province. The city contains urban city centers, urban slums, and semi-rural neighborhoods. Bukavu is highly populated with a population of >1 million [30]. The government health system in the DRC is stratified by the national level, the provincial level, the district level, health zones, and health areas or community level—from largest to smallest catchment area (Figure 1). 

### 2.2. Data Collection

Thirty-one semi-structured interviews with community members (*n* = 16) and programmatic stakeholders (*n* = 15) were conducted from April to September 2021. We developed two open-ended, semi-structured interview guides for the programmatic stakeholder and the community member participants. Interview questions—written by authors from the Université Catholique de Bukavu and Johns Hopkins Bloomberg School of Public Health—were developed with relevance to the local context and for comprehension by both child and adult participants. Questions were developed based upon the study objectives. The questionnaire also collected demographic data including gender, age, and role in household (for community member participants) (Table 1).

Programmatic stakeholder interviews were conducted in French. Stakeholders were selected using purposive sampling based on a list of stakeholders working on COVID-19 preventative activities provided by our government partners in South Kivu. We also utilized snowballing sampling during interviews with stakeholders based on individuals reported to be conducting COVID-19 preventative activities by participants. Interviews with programmatic stakeholders were conducted to learn about the successes and challenges of COVID-19 preventative response programs in the DRC. Programmatic stakeholders included health facility workers and laboratory technicians at the district level (*n* = 3); COVID-19 response committee members, health facility workers, COVID-19 surveillance officers, and members of the health department at the provincial level (*n* = 6); and workers from external developmental and non-governmental organizations (NGOs) (*n* = 6) (Table 1). 

Community member interviews were conducted in the Bukavu dialect of Swahili. Community members were from three health zones (Ibanda, Bagira, and Kadutu). These interviews explored knowledge, perspectives, and experiences with COVID-19, as well as engagement with response programs at the community level. Community members were selected using convenience sampling from slum areas in Bukavu where we have ongoing health facility and community-based surveillance. We determined the selection criteria of no functioning tap for drinking water in their home. The selection criteria are representative of urban slum areas in Bukavu which often experience water scarcity, presenting greater challenges with adhering to COVID-19 preventative hygiene measures. As a result, COVID-19 prevention programs have focused in these areas and populations. Community members included six adult males, six adult females, and four children (12 adults and 4 children) (Table 1). A parallel quantitative study observed mask wearing, handwashing, and physical distancing behaviors in indoor public spaces within the same study population (George et al., 2022, In press) [27].

### 2.3. Data Analysis

Interviews were transcribed into French. If the interview was conducted in the Bukavu dialect of Swahili, it was transcribed in Swahili and translated into French. The data analysis team comprised researchers from the Université Catholique de Bukavu and Johns Hopkins Bloomberg School of Public Health. The transcriptions were then used to develop a summary template corresponding with the interview guide; summaries and corresponding interview responses were translated into from French to English. The data analysis team wrote memos and met weekly to discuss theme development. They conducted a thematic analysis of the interview data—utilizing a hybrid inductive and deductive approach—and developed a framework of themes and subthemes. The following focal areas helped to guide theme development (deductive component): (1) community understandings of COVID-19, (2) operational successes and challenges associated with COVID-19 preventative response programs, (3) barriers to performing COVID-19 preventative behaviors, and (4) recommendations for refinement of current programs in the DRC.

The parallel quantitative observational study (George et al., 2022, In press) [27] examined COVID-19 preventative behaviors (i.e., mask wearing, handwashing, and physical distancing) and the functionality of public handwashing stations in Bukavu. This study served to triangulate our qualitative findings, augmenting the credibility of our work.

## 3. Results

### 3.1. Community Understandings of COVID-19

#### 3.1.1. Community-Level Lack of Awareness or Denial of COVID-19 Impact 

Although some community member participants were aware of COVID-19 as a disease with global impact, many were unaware or in denial of its presence and transmission in Bukavu and within their own communities. Participants explained:

*“No! I have never seen one who suffers from [COVID-19]” -Female community member, age 29 years, April 2021*

*“Since I arrived in Bukavu and Goma, I have never heard of anyone who has died [from COVID-19] either in the neighborhood or among the neighbors” -Female community members, age 20 years, April 2021*

Among the community members who were aware of COVID-19 transmission in their area, many had family, friends, or neighbors who had contracted or had passed away from the disease.

*“It arrived in this neighborhood because when one person died at the [Ciriri Hospital], the other died at the Saint Luc clinic, the other at Panzi [Hospital]” -Male community member, age 58 years, April 2021*

*“I know two or three people who died, but it was only after their death that it was said that they were coronavirus cases” -Male community member, age 33 years, April 2021*

News from local COVID-19 treatment centers sometimes alerted community members to the presence of the disease in their area:

*“Well, I don’t know anyone, and I haven’t heard about it yet, but I hear that there are people at the general hospital [COVID-19 treatment center], but there’s no one I know personally in my circle” -Female community member, age 42 years, April 2021*

#### 3.1.2. Understandings of COVID-19 Transmission Mechanisms and Origin

Knowledge of COVID-19 transmission and origin varied greatly between community members. Some correctly identified transmission pathways such as close contact with an infected person and coughing or sneezing, and could identify fever, coughing, headaches, and loss of taste and smell as symptoms of COVID-19. All community members mentioned mask wearing, physical distancing, and/or proper hygiene practices as methods of COVID-19 prevention. The elderly and individuals *“with chronic disease...with diabetes, tuberculosis, [and high] blood pressure”* (male community member, age 40 years, April 2021) were noted as higher risk for infection and experiencing more severe health consequences due to COVID-19.

One participant mentioned hearing that COVID-19 is “witchcraft sent by the Beast (666)” and a sign of the Biblical end of times. Programmatic stakeholders working at the community level also reported hearing this from community members. Other causes of COVID-19 mentioned by community members included “bad air” or miasma and dirt contaminated with the pathogen. Some community participants mentioned COVID-19 as a disease of Caucasian people and that African people were protected. Other community members mentioned hearing that COVID-19 was a moneymaking or political scheme, rather than an actual disease. One participant said:

*“People have different points of view but I know that the coronavirus exists, but others say that oooooh, the coronavirus does not exist, others say that it is a business of the Congolese, they are just looking for money” -Female community member, age 42 years, April 2021*

### 3.2. Impact of COVID-19 upon Community Members and Healthcare Workers

#### 3.2.1. Community Members

Most community members acknowledged that the COVID-19 pandemic had impacted their daily activities. Participants described severe restrictions in their movements caused by the lockdowns implemented:

*“We are like prisoners because of the coronavirus” -Female community member, age 32 years, April 2021*

The border between the DRC and Rwanda was closed in response to the pandemic, which limited travel. At the same time, concerns about COVID-19 caused fear in the community regarding traveling and being around other individuals. Community members described an overall negative economic impact of COVID-19 in Bukavu, as well as household financial consequences. Job and food insecurity arose as major challenges:

*“It blocked everything. We could no longer find food, it caused the closure of work, we could no longer get the money where we received it” -Female community member, age 17 years, April 2021*

#### 3.2.2. Healthcare Workers

Frontline healthcare workers were also heavily impacted by COVID-19, particularly at the beginning of the pandemic. Lack of necessary PPE and hygiene materials in healthcare facilities and initial uncertainty about disease transmission pathways placed workers at greater risk of infection and mortality. A health facility worker reported that multiple physicians had died from COVID-19 in the first 6 months of the disease reaching their area. The lethal toll and limited access to preventative materials lessened the morale and motivation of response teams. The refusal to practice preventative measures by patients within healthcare facilities led health providers to fear contracting COVID-19:

*“there is another one who, once at the handwashing station, turns back since he says he can’t wash with that smelly [chlorinated] water, and even if we force him he categorically refuses and since he is sick, we receive him like that and in this case we have to protect ourselves more so that he can’t pass on what he has to us” -Male district level health facility worker, age 71 years, April 2021*

### 3.3. Barriers to Performing COVID-19 Preventative Behaviors and Vaccination

#### 3.3.1. Barriers at the Community Level and in Healthcare Facilities

Denial of COVID-19′s existence throughout the community at the start of the pandemic was viewed as a major barrier to adherence to COVID-19 preventative behaviors:

*“the biggest challenge is the denial of the disease by the population, including intellectuals, there has been a lot of denial... and I found that social [media] had a lot to do with it. There have been quite a few videos or audio where white people, Americans, Europeans, Swiss, where they show that it’s a mafia, a plot to kill people” -Male district level health facility worker, age 36 years, July 2021*

Political distrust was high in the community, and some viewed the disease as *“a political set-up... [or] government scheme to make money”* (male community member, age 14 years, April 2021). Participants discussed concerns that COVID-19 was inside masks which may have been a major barrier to masking behaviors:

*“there are masks that contain COVID-19 drugs [that] put this coronavirus disease in [the mask]” -Female community member, age 29 years, April 2021*

The concern about COVID-19 inside distributed masks created a preference for use of locally manufactured masks:

*“they are afraid of some [masks] saying that those that come from White people contain... the coronavirus. There is the quality that we trust, we trust the masks made here locally” -Male community member, age 50 years, April 2021*

Programmatic stakeholders also discussed barriers to practice COVID-19 preventative measures within healthcare facilities and in the community. Firstly, the lack of available PPE and hygiene materials hindered preventative activities within healthcare facilities. Secondly, preventative measures such as handwashing with chlorinated water or wearing a mask caused discomfort or nuisance at times:

*“Well, I only see that they put it around the chin. It doesn’t cover the face, when you ask him why he doesn’t wear it, he says it chokes him. He wears it reluctantly just so he doesn’t get arrested” -Female community member, age 42 years, April 2021*

Thirdly, physical distancing was challenging on crowded buses and in markets:

*“Many have stopped wearing the masks and are walking around, others are stuck in the bus, others are packed together in the celebration rooms [e.g., places for weddings and mourning], others do not respect the handwashing anymore” -Male community member, age 38 years, April 2021*

*“in popular districts, the places like the market of Kadutu where you have between fifty and hundred thousand people who pass there per day, there was no, no [preventative] measure” -Male district level health facility worker, age 41 years, May 2021*

#### 3.3.2. Perspectives on COVID-19 Vaccination

Although COVID-19 vaccination rates were low at the time of these interviews (COVID-19 vaccination began in April 2021 [31]), several community members acknowledged the importance of vaccination in overcoming the burden and lethal toll of the disease:

*“Let them bring the vaccine, only that will help. Because if the vaccine is given even if you come in contact with people, it decreases the risk. There’s nothing else they can do because they can’t feed us without us working. To say that everyone stays at home is not possible” -Female community member, age 42 years, April 2021*

Even while recognizing the importance of vaccination, community members noted their personal hesitancies and doubts, as well as skepticism within their broader community. Some programmatic stakeholders were also concerned with vaccine hesitancy and ensuring frontline healthcare workers receive vaccines:

*“The vaccine is already there, and we will ask that research be conducted on the practical knowledge and attitudes of the community with respect to the vaccine so that vaccination can be carried out peacefully and there can be acceptance” -Male health facility worker in risk communication and community involvement, age 30 years, May 2021*

*“it is absolutely necessary to provide these structures with prevention and infection control kits to avoid that frontline staff can be affected and, secondly, vaccination as well. We are trying to ensure that all frontline staff are vaccinated in two doses to prevent them from being affected by this disease” -Male external organization worker, age 40 years, August 2021*

Hesitancy was attributed to the speed with which the COVID-19 vaccine was developed:

*“For me, the vaccine is important if you get it. But I still have doubts. It is true that the research was carried out by scientists who have a great knowledge, but they launched it on the market in a hurry because they did not take enough time to refine it. Nowadays, using it on people is good but I still have doubts if it has been well worked or not” (male community member, age 48 years, April 2021).*

### 3.4. Experiences with COVID-19 Preventative Response Programs

#### 3.4.1. Sources of COVID-19 Health Communication

Television and radio communication were often noted as sources of information about COVID-19:

*“As we are shown in television commercials, Corona manifests itself in many ways. Some people have a high fever, others say they have a headache, others have the flu and a cough. These are the symptoms by which you can identify someone who has Corona” -Female community member, age 20 years, April 2021*

Other sources of COVID-19 preventative information included posters and billboards in the community or in health facilities, word-of-mouth from healthcare providers, word-of-mouth through family and friends, and social media platforms (i.e., WhatsApp and Facebook). Direct interaction with community members for health communication (e.g., health workers speaking with residents door-to-door) and engaging religious and local leaders were considered effective measures in improving preventative behaviors. One participant said:

*“we used the religious leaders, it helped us to fight against rumors and false information that were circulating because in Africa the community trusts the priests and the different pastors or religious [leaders]” -Male health facility worker in risk communication and community involvement, age 30 years, May 2021*

Some community members preferred to be spoken to directly about COVID-19, rather than to see or hear advertisements:

*“Yes, I think it’s better to have a person who comes to educate, a person with whom to exchange, he can explain to you, he can make you understand how to protect yourself because you will exchange with him. It is better that the person comes physically” -Male community member, age 50 years, April 2021*

#### 3.4.2. COVID-19 Prevention Efforts and Activities of Foreign Organizations

Community members and programmatic stakeholders mentioned the role of foreign organizations in provision of COVID-19 preventative materials and raising awareness about COVID-19 in the community.

*“In my area, the people who came to help us with the coronavirus came... I think it was the people from [foreign organization name redacted]... They came and asked us to protect ourselves from the coronavirus by washing our hands before eating and looking for the disinfectant and mask” -Male community member, age 38 years, April 2021*

In addition to preventative material provision, foreign organizations assisted with rapid training of healthcare personnel to address COVID-19. With COVID-19 presenting barriers to meeting in-person for these trainings, external organizations met virtually with smaller groups of healthcare personnel who then trained other workers in a cascade training process. Some foreign organizations focused on engagement in the COVID-19 response at the community level. One group provided hundreds of religious leaders in several health zones with training on incorporating COVID-19 sensitization into their religious activities. They also provided the leaders with mobile phones to improve communication back with the organization.

#### 3.4.3. Compliance with and Functionality of COVID-19 Preventative Measures

Community members and programmatic stakeholders both discussed the mask mandate in public spaces. This mandate was enforced through fines and a risk of arrest which was upheld by police presence. Although it was successful in increasing compliance with mask wearing protocols, when the mandate was lifted, people reverted to widespread non-compliance. Furthermore, the mask mandate focused on any mask wearing rather than mask wearing over both the nose and mouth. One community member said:

*“I have to admit that many people respect these [mask wearing] measures because of the authorities’ constraint, but since the measures have been lifted in relation to lockdown, many have thought that the disease [COVID-19] is over” -Male community member, age 38 years, April 2021*

Participants also mentioned handwashing stations in public areas. However, these handwashing stations were sometimes not usable, lacking water or cleansing agents:

*“I have seen [handwashing stations] placed in markets, in public places, in front of stores, in front of schools, in front of hospitals, there were buckets for handwashing and they still exist.... But in the markets this measure is not respected because you will notice the presence of handwashing buckets but without water” -Male community member, age 33 years, April 2021*

One community member mentioned seeing handwashing stations and COVID-19 health communication, but the lack of follow-up with community sensitization stifled continued compliance to preventive measures in their community. 

Some community members felt disconnected from the on-the ground impact of the ongoing government COVID-19 prevention activities:

*“we hear that the authorities continue to do their [COVID-19] work but there is no visible impact.... The authorities on the official level... are doing their job but you will really find on the ground that there is some failure” -Male community member, age 38, April 2021*

#### 3.4.4. Challenges to COVID-19 Response Program Implementation

Programmatic stakeholders identified a lack of coordination between health system actors involved in the COVID-19 response. Efforts at the health zone, district, provincial, and national levels, and efforts by foreign organizations, were occurring simultaneously with limited communication between partners to coordinate activities:

*“the challenge that I can raise was the lack of organization... it seems that there was no planning, we did things without planning so that we could have many partners but... they did not put all their means together to fight [COVID-19]. So, each partner came from his side and supported his side without any [resource] pooling, that was a weakness” -Male health facility worker in risk communication and community involvement, age 30 years, May 2021*

#### 3.4.5. Setbacks and Synergies of the Ebola Virus Disease and COVID-19 Responses

Within healthcare facilities, the most pressing challenge discussed was inadequate availability and funding for COVID-19 preventive materials (i.e., masks, gloves, handwashing materials, vaccines). Ongoing Ebola Virus Disease (EVD) control exacerbated these resource restraints and teams of frontline health workers remained unpaid for their previous efforts when the pandemic began:

*“[the COVID-19 pandemic began] in a context of very limited resources, we had just had the EVD and... we were really in difficulty because even the teams that were used during the response against the EVD, we are not paid until today and [then] there was the COVID-19 that arrived” -Male provincial health department worker, age 38 years, May 2021*.

Some healthcare workers lacked proper training and preparation to address the COVID-19 burden at the start of the pandemic. Other programmatic stakeholders including healthcare workers and their teams were able to “capitalize” upon lessons learned and preparation from the ongoing nationwide EVD efforts:

*“We have capitalized on the achievements of the EVD. And so, we had actors here locally at the level of the province... there was the rapid involvement of some partners such as [foreign organization name redacted] who helped us to quickly train the actors. And that is a success. This also allowed us to train all the actors in the health zones so that we could set up rapid [COVID-19] intervention teams in each health zone” -Male provincial health department worker, age 58 years, April 2021*

The added burden of COVID-19 strained already limited resources; however, infrastructure was already in place such as handwashing stations and community mobilization strategies from EVD. Ongoing training of healthcare workers to address EVD shifted to focus on COVID-19. Close interactions between health actors at the provincial level and external organizations fostered coordinated and rapid training of provincial level healthcare personnel:

*“the province was already doing training in terms of waiting for cases of Ebola virus that were rampant in North Kivu, there was some knowledge gained in terms of [prevention and control] and this is why we relied on these measures, essentially prevention measures in terms of physical distancing, handwashing and so on”-Male provincial level health facility worker, age 41 years, May 2021*

Health facilities rapidly organized intake and triage systems. Incoming patients were required to wash their hands before entering the hospitals and then were screened for COVID-19 symptoms and potential contact with infected persons. Patients with concerning symptoms were taken to quarantine for further investigation and then isolation with confirmed or likely COVID-19 diagnosis, similarly, to approaches already in place for EVD.

### 3.5. Importance of Research and Evidence-Based Decision Making for The COVID-19 Response

Finally, programmatic stakeholders—including district, provincial, and external organization workers—discussed the importance of investing more in scientific research and allowing evidence-based decisions to drive COVID-19 response efforts. Some felt this focus was overlooked, particularly related to the monitoring and evaluation of implemented response programs:

*“the big weak link in the [COVID-19] response in the province was something that we all neglected, and that was research.... even if I have just praised the interventions we have carried out, ... we have not been able to do quality research to ensure that there has been an impact.... I think that can help us better evaluate ourselves but also guide strong interventions” -Male provincial level health facility worker, age 38 years, May 2021*

*“Research could easily guide a lot of things. But everything that was being done was being done as novices, especially since the disease is still new, but if scientists are also brought to the forefront, I think that they could help to re-frame, to direct a lot of things” -Male external organization worker, age 50 years, July 2021*

A few stakeholders mentioned political interests rather than scientific evidence driving decision making:

*“the people who manage COVID are first the politicians. So it’s the politicians, it’s the president, it’s the governor, it’s the minister and so the challenge is that as a scientist at times there are political decisions and you have a scientific orientation... every time you had to convince them, sometimes they weren’t convinced, sometimes they did what they had to do without your consent or... without asking for your opinion” -Male provincial level health facility worker, age 50 years, May 2021*

Programmatic stakeholders also identified a need to address COVID-19 denial, noncompliance to COVID-19 preventative measures, and vaccine hesitancy with transdisciplinary approaches:

*“I think that it would be better... to associate different disciplines to understand the behavior of people in relation to the disease... it is medicine, anthropologists, sociologists, psychologists... who can get together and study the behavior of our population specifically in relation to the disease because if we understand why people in our country have refused the disease, we can even understand why there is denial of vaccines and how to respond” -Male district level health facility worker, age 38, June 2021*

One participant mentioned the importance of research in optimizing the efficiency and effectiveness of COVID-19 response initiatives, as well as leveraging strengths and capacities of the South Kivu province and in communities:

*“We are a province that abounds in unquantifiable resources, high-level human resources, with young researchers, visionaries, young epidemiologists like ourselves” -Male provincial level health facility worker, age 36 years, July 2021*

We have summarized our key findings in Table 2. We stratified our findings between the following levels: health system/structural, programmatic, community, and individual. We have also differentiated between factors that arose in the interviews which pertain to overall disease prevention and to COVID-19 preventative activities specifically.

## 4. Discussion

Our study explored community understanding of COVID-19 and disease prevention, experiences of community members and healthcare providers during the COVID-19 pandemic, and successes and challenges of COVID-19 preventative programs in South Kivu, DRC. Some community members attributed COVID-19 to actions of malevolent neighbors, miasma (“bad air”), or spirits. Awareness of COVID-19 preventative measures was high, largely because of radio and TV health promotion programs. Concurrent prevention and control of EVD prepared healthcare workers to mobilize existing resources and put preventative measures in place to address the emergent COVID-19 pandemic [32,33,34]. 

Consistent with previous studies in Ethiopia and Uganda, in our current study most participants were aware of COVID-19, even if they did not know of its presence in their neighborhood [18,35]. Community members conveyed mistrust of COVID-19 health communication and concerns that COVID-19 was a political or moneymaking scheme. COVID-19 denial and perceived invisibility of COVID-19 transmission were major barriers to the success of preventative programs at the community level. This finding is also similar to previous studies in Côte d’Ivoire and Nigeria which found political distrust and widespread rumors about COVID-19 to be major barriers to response efforts [20,21]. Similar to our findings, during the 2018–2019 EVD outbreak in DRC, low institutional trust and widespread misinformation were associated with reduced adoption of EVD preventive behaviors [36]. These findings demonstrate the importance of engaging communities in COVID-19 preventative programs in our study setting to ensure intervention approaches are tailored to the community context.

Engaging community and religious leaders in COVID-19 prevention programs was viewed as a critical component of building trust and dispelling rumors and misinformation in our study setting. This is consistent with findings observed in Zimbabwe, Nigeria, and Ethiopia [15,18,19]. Furthermore, COVID-19 response programs delivered in-person, allowing for questions and discussions, were considered by community members more engaging than written content such as posters and billboards. Therefore, future COVID-19 response programs should promote this approach in our study setting when feasible and with proper PPE. While social media sites (e.g., WhatsApp and Facebook) can be sources of COVID-19 misinformation, they should also be leveraged to provide factual and context-specific health communication and have the benefit of reaching a larger audience at minimal cost [12,14,37,38,39,40]. A multi-prong approach that includes in-person visits, social media, and radio and television will likely be needed for COVID-19 preventative program delivery in our study setting.

Inadequate access to PPE (e.g., hygiene materials) and lack of WASH infrastructure (e.g. running water for handwashing) presented major barriers to preventative behaviors in healthcare facilities and in the community [13,16,22]. Community participants mentioned that the handwashing stations in public places often lacked water and cleansing agents. This is consistent with our parallel quantitative study which found that the majority (61%) of handwashing stations in indoor public spaces (e.g., entrances of health facilities, schools, and universities) in Bukavu did not have both water and handwashing agents present (George et al., 2022, In press) [27]. Community residents interpreted the lack of upkeep of handwashing stations and relaxing of masking mandates as meaning that COVID-19 transmission was less of a concern. Therefore, promoting positive health behavior change at the community level requires maintaining availability and accessibility of preventative materials. 

Community members and programmatic stakeholders mentioned that adherence to mask wearing was high during police enforcement, and after police enforcement subsided, so did mask wearing. Even during enforcement of the mask mandate, it was noted that individuals were often not wearing their mask over both their nose and mouth. This is also consistent with our quantitative study which found that only 16% of >4600 observed individuals wore a mask completely covering their nose and mouth in indoor public spaces (George et al., 2022, In press) [27]. Engaging local leaders (e.g., religious leaders, community healthcare workers, and school officials) in raising awareness about mask wearing can help to increase compliance. Locally made masks are also likely needed to increase community confidence in the safety of masks. Programs training community members to make their own masks have been implemented in many settings in sub-Saharan Africa approach [41]; this is one promising approach. Furthermore, the broader context of political and civil unrest and community distrust of government authorities makes police enforcement of mask wearing challenging. One potential alternative would be the model implemented already in some communities of Bukavu and Goma, DRC where communities appoint individuals to serve as part of a community watch program and hire military personnel directly to ensure mask wearing in public spaces [24].

Community members and programmatic stakeholders discussed interest in COVID-19 vaccination. However, community members expressed concerns about taking the COVID-19 vaccine, and skepticism regarding widespread acceptance of the COVID-19 vaccine. Previous studies in DRC have identified COVID-19 vaccine hesitancy and low prioritization of obtaining the vaccine considering other life demands as a significant barrier to vaccine use [16,42]. Through COVAX, the DRC received 1.7 million doses of the AstraZeneca COVID-19 vaccine. Denial of this vaccine by several European countries, public rebuke from the DRC president, and aforementioned community vaccine hesitancy caused very low uptake and the majority of these doses were returned before expiry [43]. Vaccine uptake may be improved by conducting qualitative research regarding understandings and attitudes towards the COVID-19 vaccine and engaging local and religious leaders to build trust at the community level. Future research should identify the drivers of vaccine hesitancy and perspectives of COVID-19 vaccination in South Kivu communities. 

Programmatic stakeholders noted the lack of coordination for COVID-19 response programs between partners at the health zone and provincial levels. A range of actors were involved and disorganization at multiple levels complicated preventative efforts. For example, a participant noted that partners working within the same community were unaware of their respective roles in response efforts which created confusion and conflicting activities. Multisectoral and transdisciplinary teams would improve coordination, and these teams can better address the multifaceted nature of the COVID-19 pandemic.

The EVD outbreaks in the DRC highlighted the importance of surveillance and contact tracing, community-level sensitization and promotion of preventative activities, isolation and quarantine of confirmed and suspected cases, and overall health system strengthening [8,44,45]. However, investments and efforts to limit EVD transmission have left an environment of limited resources and a strained health workforce [46]. For example, one participant explained response teams previously addressing EVD had not received financial compensation until after COVID-19 activities had already begun. Nevertheless, the overlapping strategies to limit EVD transmission laid foundations for addressing COVID-19, as noted by programmatic stakeholders and in the literature [8,33,44,45]. Standard operating procedures, hygiene materials, and rapid response teams from EVD programs were adapted to confront emergent COVID-19 [33]. Health facilities quickly established separate centers for intake and triage of potential COVID-19 cases, similarly to systems of diagnosis and quarantine for EVD. Community healthcare workers played a significant role in implementing community-based surveillance (i.e., screening, rapid diagnostic testing, and contact tracing) in both EVD and COVID-19 response [33]. As we expect more novel infectious diseases to emerge globally, the COVID-19 prevention response effort in the DRC serves as a model for leveraging existing health infrastructure and measures to new challenges.

Producing and prioritizing transdisciplinary research was also considered by programmatic stakeholders as a necessity moving forward. The lack of investment in research regarding COVID-19, including health behavior at the community level, and monitoring and evaluation of response programs was seen as a significant oversight and hinderance to improving program effectiveness. The local reality of unrest and political distrust alongside stigmatization and fear of disease from EVD outbreaks produces an challenging context for health communication. Developing effective social and behavior change communication requires an additional sociological and historical lens. These capacities are already present in South Kivu, however are not utilized in current strategies. Building upon and uplifting existing scientific, sociological, and behavioral research capacities in the areas where health programs are implemented is an effective way to prioritize evidence-based decision making and contribute to the decolonization of global health research—a pressing and ongoing global imperative [47,48,49,50].

Vaccination is undoubtedly a critical element of population-level COVID-19 prevention, as described by study participants and previous studies [51]. However, vaccination alone is not enough to curb COVID-19 transmission. Mask wearing, physical distancing, and hygiene practices must be implemented concurrently to lessen the spread of COVID-19 [52,53,54,55]. Logistical challenges with vaccine distribution, inequities in vaccine access, and widespread vaccine hesitancy—as evidenced in this study—hinder COVID-19 vaccine utilization, emphasizing the importance of concurrent non-pharmaceutical methods of prevention [43,56,57,58,59]. Holistic community-centered [60] strategies integrating vaccination, surveillance, WASH, and other non-pharmaceutical preventative methods must lie at the forefront of the COVID-19 response in South Kivu.

We have developed several recommendations for future COVID-19 preventative programs which are applicable at the provincial and national levels (Figure 1). We recommend to:

1. Prioritize trust building from the national level to the community level: Political distrust arose as an important factor related to COVID-19 denial and compliance with preventative measures. Developing and employing strategies to bolster community level trust is imperative to behavior change related to COVID-19 prevention.
2. Use community-developed strategies to encourage compliance with preventative measures (e.g., community watch programs and community hired military personnel to enforce mask wearing): Engaging community in the development of COVID-prevention can increase compliance with preventative strategies;
3. Utilize local capacities for research and developing prevention materials to improve community trust: Disease prevention, behavior change, and health communication require multisectoral and transdisciplinary perspectives. Capacities for transdisciplinary research already exist in South Kivu. Local researchers can provide emic points of view that foster community trust and contribute to effective interventions.
4. Capitalize upon ongoing disease prevention strategies to confront novel, emergent diseases (i.e., EVD → COVID-19): Previous EVD outbreaks hindered COVID-19 prevention due to resource limitations, however adaptable strategies for prevention and control were able to be leveraged from EVD to confront the additional COVID-19 burden. Existing programs, strategies, and infrastructure should be adapted to address emergent diseases.

This study has a few limitations. First, all interviews were conducted in South Kivu which limits transferability to other provinces in eastern DRC. Future similar studies are needed in other areas of eastern DRC. Secondly, future formative research should include interviews with health providers at more levels including community health workers. Community health workers have more insight into the programmatic aspect of the COVID-19 response at the community level. *Banda*

The strengths of this qualitative study are, firstly, that we interviewed adult and child community members in different health zones. Programmatic stakeholders interviewed included healthcare workers at the provincial and district levels, as well as external organization workers. Secondly, our qualitative findings complement the quantitative study run in parallel to this one, allowing for a multiple method approach to evaluate COVID-19 preventative programs. Finally, our developed recommendations are transferable to other settings globally.

## 5. Conclusions

**Figure 2:**
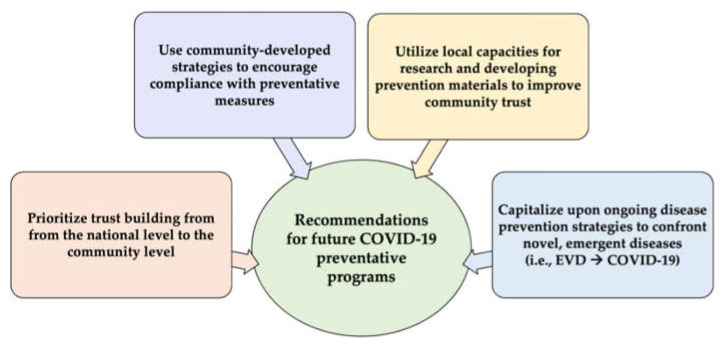
Recommendations for developing the continued COVID-19 response in South Kivu, DRC.

Our study provided insights into community perspectives of COVID-19, the impact of the pandemic within the community and in healthcare facilities, barriers to practicing preventative measures, and successes and challenges of COVID-19 preventative programs in South Kivu. Through this study we developed recommendations for delivery of COVID-19 preventative programs (Figure 2). These recommendations may be relevant to other settings in sub-Saharan Africa where fragile health systems and public health infrastructure make delivery of COVID-19 preventative response programs challenging. Similar studies are needed to tailor COVID-19 preventative programs to the local context in other settings globally.

## Figures and Tables

**Table 1:** Semi-structured interviews conducted with community members (*n* = 16) and programmatic stakeholders (*n* = 15).

Community Member Interviews (April 2021)	Paternal Member of Household (33–58 Years)	Maternal Member of Household (29–42 Years)	Child in Household (12–17 Years)	Total
Explore community understandings, knowledge, and experiences with COVID-19	6	6	4	16
Understand community engagement with and perspectives on COVID-19 response programs				
**Programmatic Stakeholder Interviews (April to September 2021)**	**District Level Participants**	**Provincial Level Participants**	**External Organization Participants**	**Total**
Understand successes, challenges, and experiences with COVID-19 hygiene preventative response programs from programmatic stakeholders	3	6	6	15

**Table 2:** Summary of key findings from semi-structured interviews with both community member and programmatic stakeholder participants. Findings have been organized by level of influence (health system/structural, programmatic, community, and individual), as well as pertinence to overall disease preventative activities or specifically to COVID-19 preventative activities.

	Factors Related to Overall Disease Prevention	Factors Specific to COVID-19 Preventative Activities
**Health system/Structural**	⇒ Lack of coordination and organization of activities between health system levels ⇒ Political priorities conflicting with scientific evidence	⇒ COVID-19 prevention and control policies (i.e., mask mandates and enforcement, lockdowns, travel restrictions)
**Programmatic**	⇒ Lack of coordination and communication between prevention program actors ⇒ Foreign organization assistance in resource provision, community outreach, and training of healthcare workers ⇒ Inadequate access to and funding for personal protective equipment and hygiene materials ⇒ Non-functional communal handwashing stations ⇒ Limited resources due to ongoing EVD response ⇒ Minimal monitoring and evaluation of implemented prevention programs	⇒ Capitalizing upon Ebola Virus Disease prevention infrastructure and training protocols ⇒ Need for transdisciplinary research ⇒ Patient non-compliance with COVID-19 preventative measures in health facilities ⇒ Decreased healthcare worker morale/motivation
**Community**	⇒ Financial hardship ⇒ Food and water insecurity ⇒ Political distrust	⇒ Knowledge of COVID-19 transmission in local area ⇒ COVID-19 denial at the community level ⇒ Non-compliance with COVID-19 preventative measures ⇒ COVID-19 vaccine skepticism and hesitancy ⇒ COVID-19 health communication via social media ⇒ COVID-19 misinformation spread via social media ⇒ Difficulty with physical distancing in crowded buses and markets
**Individual**	⇒ Disturbed by smell of chlorinated water	⇒ Knowledge of COVID-19 preventative measures ⇒ Mask wearing, physical distancing, handwashing ⇒ Improper mask wearing (i.e., only over mouth or underneath chin) or refusal to wear mask ⇒ Understanding of COVID-19 etiology/causation ⇒ Transmission pathways, signs and symptoms, origin, at-risk groups ⇒ Discomfort/nuisance when wearing masks ⇒ Trust in locally manufactured masks and sanitizers ⇒ Concern about COVID-19 inside of masks

## Data Availability

The data supporting the conclusions of this article are available from the corresponding author upon reasonable request.
